# Clinical assessment of standing and gait in ataxic patients using a triaxial accelerometer

**DOI:** 10.1186/s40673-015-0028-9

**Published:** 2015-08-06

**Authors:** Akira Matsushima, Kunihiro Yoshida, Hirokazu Genno, Asuka Murata, Setsuko Matsuzawa, Katsuya Nakamura, Akinori Nakamura, Shu-ichi Ikeda

**Affiliations:** Department of Neurology and Rheumatology, Shinshu University School of Medicine, 3-1-1 Asahi, Matsumoto, 390-8621 Japan; Division of Neurogenetics, Department of Brain Disease Research, Shinshu University School of Medicine, 3-1-1 Asahi, Matsumoto, 390-8621 Japan; Kissei Comtec Co., Ltd, 4010-10 Wada, Matsumoto, 390-1293 Japan; Division of Clinical and Molecular Genetics, Shinshu University Hospital, 3-1-1 Asahi, Matsumoto, 390-8621 Japan; Intractable Disease Care Center, Shinshu University Hospital, 3-1-1 Asahi, Matsumoto, 390-8621 Japan

**Keywords:** Spinocerebellar ataxia, Multiple system atrophy, Triaxial accelerometer, Gait analysis, SARA

## Abstract

**Background:**

The aim of this study was to investigate the usefulness of a triaxial accelerometer for the clinical assessment of standing and gait impairment in ataxic patients quantitatively. Fifty-one patients with spinocerebellar ataxia (SCA) or multiple system atrophy with predominant cerebellar ataxia (MSA-C) and 56 healthy control subjects were enrolled. The subjects, with a triaxial accelerometer on their back, were indicated to stand for 30 s in four different conditions (eyes opened or closed, and feet apart or together) and then to walk 10 m for a total of 12 times on a flat floor at their usual walking speed. In standing analysis, the degree of body sway was assessed. In gait analysis, gait velocity, cadence, step length, step regularity (auto-correlation coefficient: AC), step repeatability (cross-correlation coefficient) and the degree of body sway (The ratio of root mean square in each direction to the root mean square vector magnitude: RMSR) were evaluated.

**Results:**

The degree of body sway in each standing condition and all parameters in gait showed a significant difference between the patients and control subjects. The AC and RMSR values, as well as the Scale for the Assessment and Rating of Ataxia score, showed a strong correlation with disease duration.

**Conclusions:**

Various parameters obtained by a triaxial accelerometer can be sensitive and objective markers for the assessment and follow-up of standing and gait impairment in ataxic patients.

## Background

Spinocerebellar ataxia (SCA) and multiple system atrophy with predominant cerebellar ataxia (MSA-C) present mainly with gait or truncal ataxia [1, 2]. There are no reliable biomarkers available for the assessment of cerebellar ataxia; therefore, scales based on neurological examinations such as the International Cooperative Ataxia Rating Scale or Scale for the Assessment and Rating of Ataxia (SARA) have been used widely for this purpose [3, 4].

SARA is easy to perform, but is not sufficiently sensitive for the assessment of short-term interval changes or of patients in the early stage of disease. In some cohort studies for SCA, the SARA scores changed by at most 1 or 2 points a year [5–7]. If a more sensitive and objective method of assessment is available, it will be of great value for clinical trials of new drugs or an intervention by intensive rehabilitation, because such clinical applications need a short-term assessment of their effectiveness.

For quantitative analysis of motor function, the usefulness of a triaxial accelerometer has been shown [8, 9]. A triaxial accelerometer is a small and light device that is not stressful for the subject, and can provide various useful parameters [8–13]. This device has been applied to patients with some neurodegenerative disorders such as Parkinson’s disease and Huntington’s disease [11, 14–16]. However, there is no detailed report on the analysis of standing and gait in patients with SCA and MSA-C. The aim of this study was to validate the usefulness of a triaxial accelerometer for the assessment of standing and gait impairment in ataxic patients.

## Results

There was no significant difference for the gender and age distribution between the patients and controls (gender: *p* = 0.761, age: *p* = 0.200). Disease duration, SARA score and disease subtype for the patients enrolled are summarized in Table 1.

**Table 1:** Characteristics of the subjects

	Patients	Controls
	*n* = 51	*n* = 56
	Mean ± SD (range)	Mean ± SD (range)
Male/female, n	24/27	28/28
Age, years	60.3 ± 10.4 (39–79)	57.2 ± 14.1 (31–85)
Disease duration, years	8.7 ± 6.5 (0–24)	.
SARA score (total)	8.6 ± 3.6 (1–16)	
SARA score (gait + stance)	3.9 ± 2.0 (0–8)	
Disease subtype		
SCA1	1	
SCA2	1	
SCA3/MJD	1	
SCA6	9	
SCA31	13	
ADCA^a^	9	
CCA	9	
MSA-C	7	
Ataxia associated with Hashimoto’s disease	1	

^a^Family history was supportive of autosomal dominant cerebellar ataxia (ADCA), but genetic testing was not performed

### Standing assessment

**Fig. 1:**
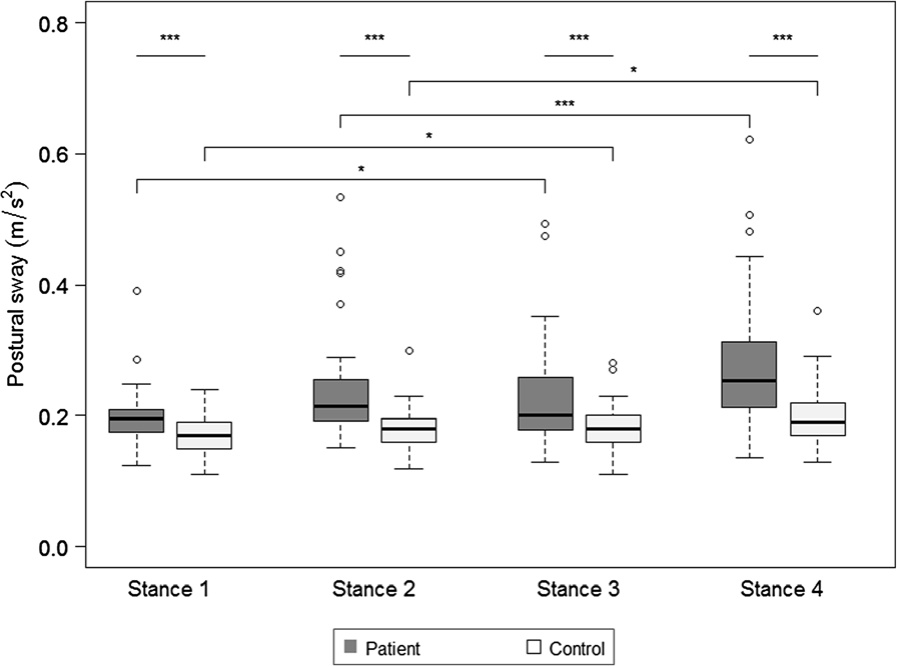
Boxplot of the postural sway in each stance with whiskers of 1.5 IQR. The circles are outliers. *p < 0.05, **p < 0.001, ***p < 0.0001

For standing, all 51 patients could maintain a standing position in Stance 1 (the feet were apart and the eyes were open), but 4 patients in Stance 2 (the feet were together and the eyes were open), 2 patients in Stance 3 (the feet were apart and the eyes were closed), and 14 patients in Stance 4 (the feet were together and the eyes were closed) could not maintain the standing position concerned. In all four stances, the horizontal acceleration vector magnitude (the postural sway) of the patients was significantly greater than that of the control subjects. The postural sway with eyes closed in each stance was significantly greater than that with eyes opened in both the patients and control subjects (Fig. 1).

### Gait assessment

**Fig. 2:**
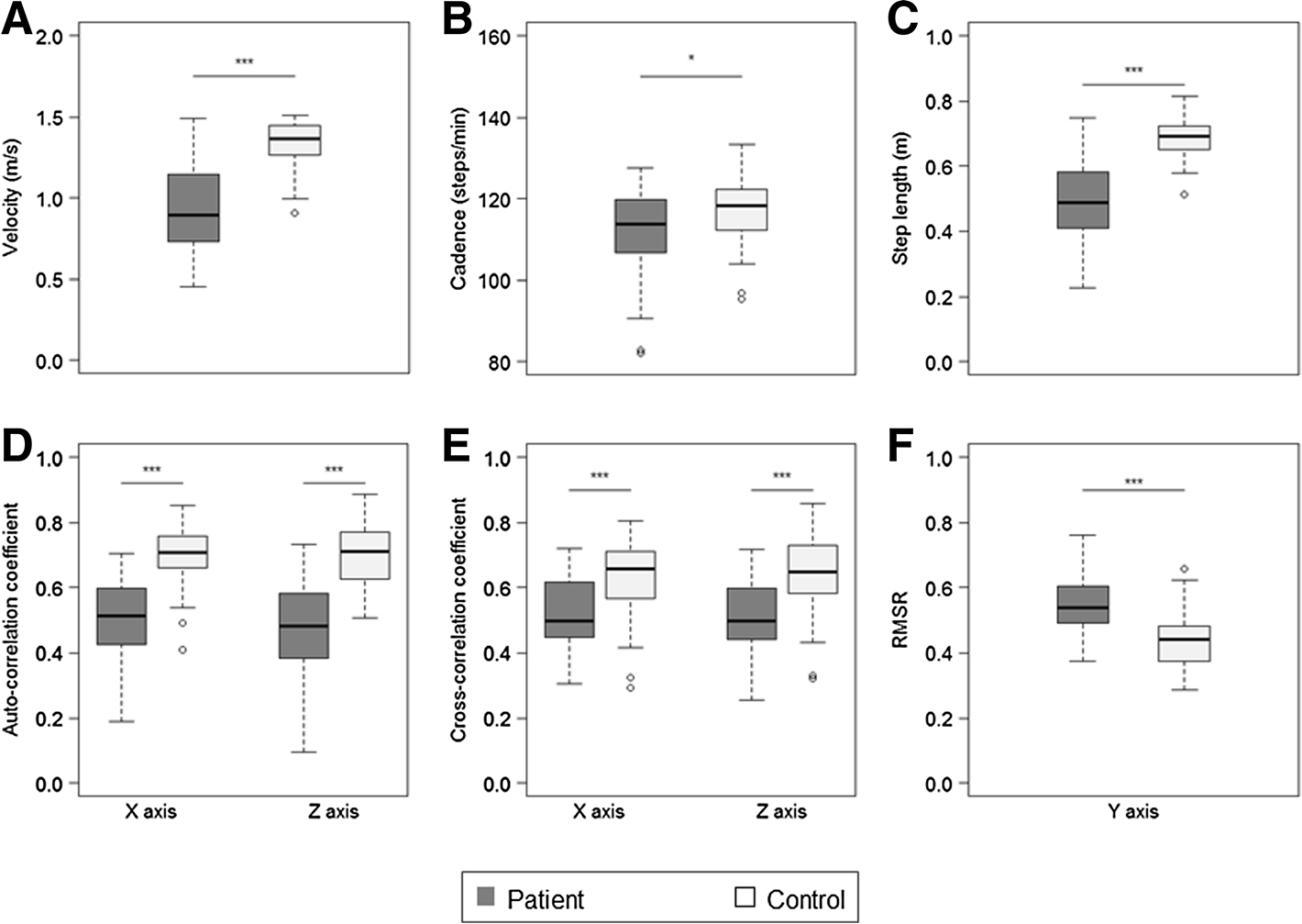
Boxplot of the parameters of gait analysis with whiskers of 1.5 IQR. **a** Velocity. **b** Cadence. **c** Step length. **d** Auto-correlation coefficient (AC) in the X and Z axis. **e** Cross-correlation coefficient (CC) in X and Z axis. **f** The ratio of root mean square in each direction to the root mean square vector magnitude (RMSR) in the Y axis. The circles are outliers. *p < 0.05, **p < 0.001, ***p < 0.0001

For gait analysis, 3 patients could not complete the 10-m trial 12 times, therefore, the cross-correlation coefficient (CC) could not be calculated for them. The auto-correlation coefficient (AC) and the CC in the left-right direction (Y axis) were not assessed because the acceleration data in the Y axis were not sufficiently sensitive to detect gait cycle and it was not appropriate to apply to the auto-correlation method. The ratio of root mean square in each direction to the root mean square vector magnitude (RMSR) in the anterior-posterior direction (X axis) and the vertical direction (Z axis) were not assessed because the RMSR was considered to have an effective meaning only in the Y axis [17]. All parameters measured in the test were significantly different between the patients and control subjects. The level of significance was very high for all parameters, except cadence (Fig. 2).

### Correlation with disease duration

The total SARA score correlated well with disease duration (Table 2). In standing, the postural sway correlated with disease duration only in the Stance 1. In gait, the AC in the X axis and the RMSR in the Y axis, as well as gait velocity and step length, were significantly correlated with disease duration (Table 2).

**Table 2:** Spearman's rank order correlation between disease duration and each parameter

	*rho* value	*p* value
SARA (total)	0.35	0.012
Stance (postural sway)		
Stance 1	0.31	0.025
Stance 2	0.27	0.070
Stance 3	0.24	0.096
Stance 4	0.18	0.310
Gait		
Velocity	-0.35	0.012
Cadence	-0.13	0.369
Step length	-0.34	0.011
AC in X axis	-0.29	0.037
AC in Z axis	-0.25	0.081
CC in X axis	-0.14	0.330
CC in Z axis	-0.21	0.161
RMSR in Y axis	0.51	<0.001

### Chronological change of the parameters

**Fig. 3:**
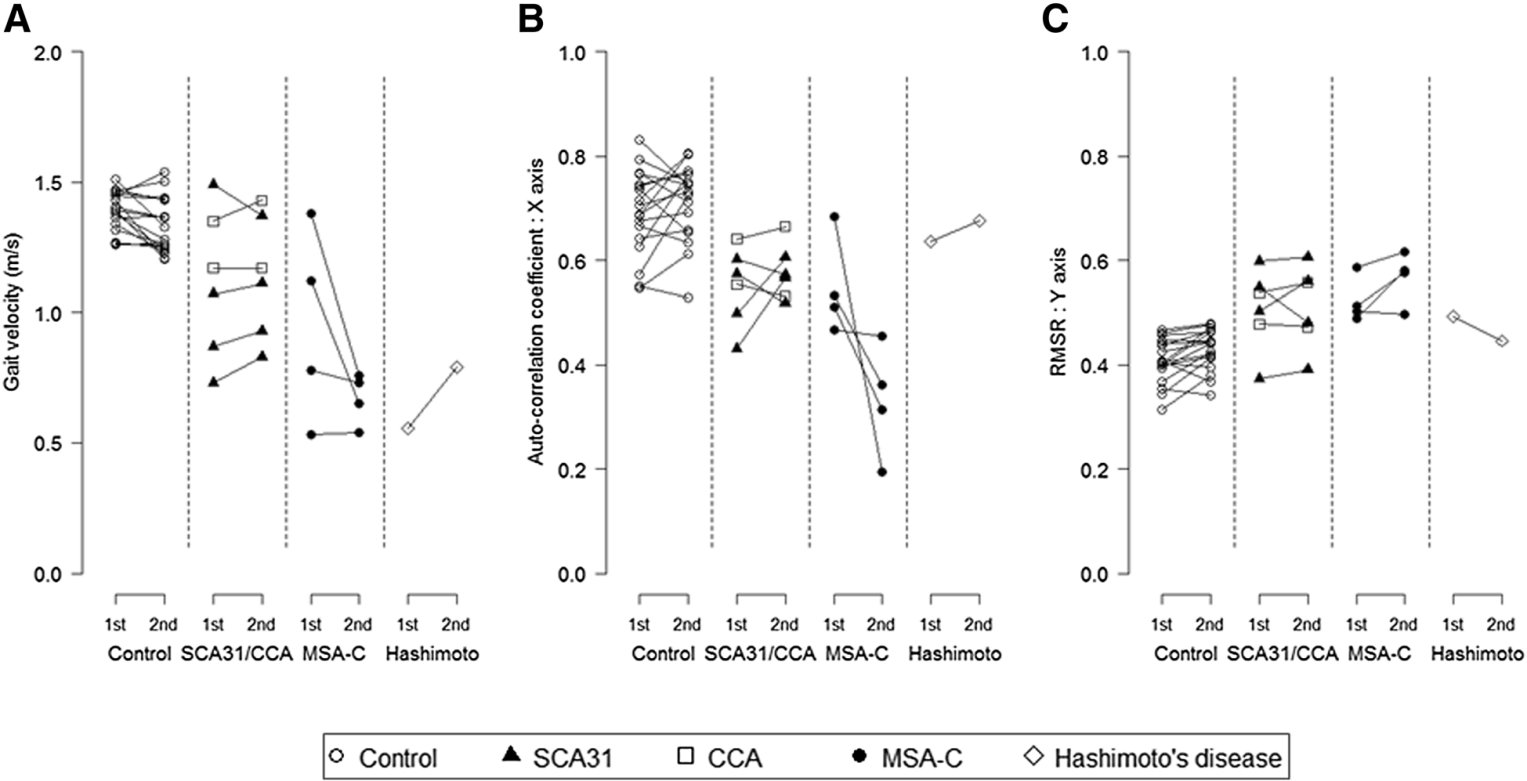
Chronological changes of the patients and control subjects. **a** Gait velocity. **b** Auto-correlation coefficient in the X axis. **c** The ratio of root mean square in each direction to the root mean square vector magnitude (RMSR) in the Y axis

Eleven patients (spinocerebellar ataxia type 31 [SCA31], 4; cortical cerebellar ataxia [CCA], 2; MSA-C, 4; ataxia associated with Hashimoto’s disease, 1) and 18 control subjects were measured twice, with an interval of approximately 6 months (Fig. 3). The patient with ataxia associated with Hashimoto’s disease was measured several times before and after the initiation of therapy (methylprednisolone pulse and oral corticosteroid). For the patients, gait velocity, the AC and the RMSR were evaluated because they were significantly correlated with disease duration. Compared with the control subjects, the AC values showed deterioration in all MSA-C patients. For the patients with CCA and SCA31, the deterioration in each parameter was not so significant. Conversely, the patient with ataxia associated with Hashimoto’s disease showed an improvement in RMSR at 1 month after the initiation of therapy. The SARA score of gait deteriorated by 1 point in one MSA-C patient, while it improved by 1 point in Hashimoto's disease patient. The SARA score of gait was unchanged in the other patients over a period of approximately 6 months.

## Discussion

Some devices such as force plates or stabilometer have been used for the quantitative evaluation of ataxic patients [18, 19]. Compared with those devices, a triaxial accelerometer has several advantages: it is much smaller, easy to handle, and does not interfere with the movement of the subject or require much time for taking measurements. Therefore, it is suitable for repeated measurements to assess the chronological changes. In addition, a triaxial accelerometer can measure throughout a three-dimensional space and gain much more parameters regarding motor function than force plates or a stabilometer.

In this study, we enrolled only ambulatory patients with cerebellar ataxia who could stand or walk by themselves (approximate total SARA score < 15). Therefore, they were relatively mild in the severity or at an early stage of ataxia, although the disease subtypes were variable. There is no curative therapy for SCA or MSA-C. Any disease-modifying intervention, if available in the future, will be more effective in the earlier stage of disease. Therefore, a method will be needed to detect subtle changes of the degree of ataxia in patients in the early stage.

The data obtained during standing assessment showed a significant difference between the patients and control subjects in all stances. In addition, the postural sway was more severe with eyes closed than with eyes open, and the degree of difference was more conspicuous in the patients than in the control subjects. More importantly, the triaxial accelerometer could detect a clear difference between the patients and control subjects even in the easiest standing task (Stance 1). These findings were basically the same as those obtained using a stabilometer [18], but a triaxial accelerometer can be applied more easily to patients who are unable to walk at all, but only can stand without any support.

In gait assessment, gait velocity and step length were significantly different between the patients and control subjects, but these parameters were probably affected by several factors other than cerebellar ataxia including the age, gender, and height of the subject [19]. Therefore, parameters other than velocity or step length will be needed to evaluate gait impairment. For this purpose, AC, CC, and RMSR were calculated and assessed in this study. Here we proposed CC as the original parameter of gait. The CC showed the degree of similarity in every 6 pairs of the walking trials from the definition. Therefore, we thought the CC represented gait repeatability. We found a significant difference in the CC, as well as in the AC and the RMSR, between the patients and control subjects. Although further study is needed to confirm the meaning of the CC in more detail, these parameters obtained by the triaxial accelerometer could be excellent markers for assessing gait disturbance in ataxic patients.

The correlation between the SARA score and disease duration in ataxic patients was confirmed previously [4, 20], and such a correlation with disease duration was also observed in the parameters obtained using the triaxial accelerometer in this study. The AC (parameter for step regularity) in the X axis and RMSR (that for the degree of sway) in the Y axis values were significantly correlated with disease duration. These results suggested that step regularity in the anterior-posterior direction and the degree of sway in the left-right direction accounted for the deterioration with disease duration. The efficacy of the AC as a parameter for differentiating patients with Parkinson’s disease from normal subjects has been reported [11, 15], which was confirmed for patients with cerebellar ataxia in this study. Furthermore, the correlation with disease duration was also found in the postural sway in Stance 1 (the feet were apart and the eyes were open), which was completed by all the patients. Although the degree of correlation in the postural sway was not as much as other parameters, the postural sway could be one of the useful parameters, especially for patients who could not walk.

We suppose that the AC and the RMSR reflect the chronological change of disease severity effectively. In the patient with Hashimoto’s disease, her subjective complaint improved after therapy. Consistent with this, this improvement was detected objectively by the triaxial accelerometer. Her AC value was unchanged by therapy because it was initially similar to the values observed in the control subjects, but her RMSR value improved. In the patients with MSA-C, the AC values deteriorated greatly during the follow-up period, indicating that step regularity of the patients was affected progressively. The SARA score of gait for a patient with MSA-C increased during the follow-up period by 1 point, but the score of the other MSA-C patient did not change. This suggests the possibility that the AC obtained by a triaxial accelerometer is a more sensitive indicator to detect the progression of gait ataxia, although the factors affecting the AC value remain unclear. In contrast, the AC value did not deteriorate in the patients with SCA31 and CCA. Although the natural history of SCA31 and CCA has been not revealed yet, both diseases are considered to have a much more protracted clinical course than MSA-C. The difference of the AC value between MSA-C and SCA31/CCA patients is very likely to reflect that of the rate of progression between these diseases. As the number of patients in each disease subtype who were measured twice was small, the comparison between the subtypes might be preliminary. Further study of the chronological changes in the AC and RMSR value is needed to see the difference of progression between the subtypes with cerebellar ataxia.

We have to state that there were some limitations in this study. Firstly, we did not assess the AC and CC in the Y axis probably because of a low sensitivity to detect gait cycle in some subjects. One of the reasons for this would be the lower sampling rate of the instruments we used. The instrument which had higher sampling rate (128Hz) was used in the report which succeeded at obtaining the AC value in the Y axis [12]. Secondly, we did not assess well how the walking speed affected on other parameters. Although the relationship between walking speed and AC was unclear [12], that between walking speed and the root mean square of acceleration in gait was clarified [9, 13]. To minimize the effect of gait velocity on measured accelerations as much as possible, RMSR was proposed [17]. It would preferable that the result was compared between the groups with the same walking speed to minimize the effect of walking speed much more, but it was difficult because the slowness of walking speed itself was just a symptom in ataxic patients. RMSR was originally used to compare hemiplegic patients with healthy subjects [17], but the method may be applicable to patients with other diseases in principle.

## Conclusion

Not only gait velocity and step length but also the parameters obtained with the triaxial accelerometer clearly discriminated the ataxic patients from the control subjects. Among several parameters, the AC and RMSR in gait analysis showed a strong correlation with disease duration, and possibly changed chronologically along with disease progression. A triaxial accelerometer can be an excellent tool for the objective and quantitative evaluation of cerebellar ataxia.

## Methods

### Subjects

Fifty-one patients clinically diagnosed with SCA or MSA-C and 56 control subjects without gait impairment were enrolled in this study (Table 1). All patients could stand and walk by themselves, but some used a cane or walker to avoid fall. Patients whose SARA score of gait was more than 6 points or SARA score of stance was more than 3 points were excluded, and patients who had a comorbid condition with disorders such as cerebrovascular or orthopedic disorders, that affects motor function were also excluded. The group of patients included a female patient with Hashimoto’s disease who complained mainly of unsteadiness on standing and gait. She was diagnosed previously as SCA, but we considered her ataxic symptoms were associated with Hashimoto’s disease based on her responsiveness to steroid therapy. Some subjects (11 patients and 18 control subjects) were measured twice with an approximately 6-month interval to evaluate chronological changes.

When the subjects were recruited, they were provided with all necessary information about the study and informed consent was obtained from all subjects. This study was approved by the Ethics Committee of Shinshu University School of Medicine (No. 2667).

### Instrumentation

A triaxial accelerometer (Jukudai Mate; Kissei Comtec Co., Ltd., Matsumoto, Japan) was used. The device was small (size, 55 mm × 80 mm, thickness, 10 mm), light (weight, ~90 g) and easy to handle. It had a sampling rate of 20 Hz. The range of detection was between -10G and +10G (G: acceleration of gravity, 1G = 9.80665m/s^2^) and the resolution power was 0.02G. The data acquired by the device were analyzed by BIMUTAS II (Kissei Comtec Co., Ltd., Matsumoto, Japan) which was developed for biological processing.

### Measurements

The device was attached to the back and the median of L3 of the subject by an elastic belt. SARA was evaluated by the first or second author on the same day of the measurements. The measurements were taken in the following order by more than three examiners. One of the examiners was responsible only for time keeping, while the others stood or walked along with the subject to prevent fall.

#### Standing

In the assessment for standing, the subjects were indicated to lower their arms to the sides of their body and to stand for 30 s. The test was stopped when one foot or both feet moved. The assessment was performed serially in the following four different conditions: (1) the feet were placed 25 cm apart and the eyes were open (Stance 1); (2) the feet were placed together and the eyes were open (Stance 2); (3) the feet were placed 25 cm apart and the eyes were closed (Stance 3); and (4) the feet were placed together and the eyes were closed (Stance 4). The subjects who were not able to stand for 30 s during a stance were excluded from the analysis of that stance.

#### Gait

After the measurement of standing, the subjects were asked to walk on a flat floor at a speed they were comfortable with. The walking distance was 10 m. The walking test was repeated 12 times (6-times reciprocating walk) consecutively. For subjects who found it difficult to walk 12 times, the test was aborted at the end of the third reciprocating walk. All subjects were instructed to stop walking at 3 m beyond the end of the walkway during each 10-m test. As the patients were often unstable at the beginning and end of walking [21], the data at the beginning and end of the test were eliminated and those for 6.4 s from the middle part were used for gait analysis.

### Parameters

The axes of the direction of acceleration were defined as follows: X axis, anterior-posterior (front-back) direction; Y axis, left-right (crosswise) direction; and Z axis, vertical (head-foot) direction.

#### Standing

The degree of body sway in standing, as the horizontal acceleration vector magnitude (postural sway) was calculated every 0.05 s. The mean value of the postural sway for 30 s was used for the analysis, which represented the degree of sway during standing.

#### Gait

The following parameters were taken: (a) velocity, (b) cadence, (c) step length, (d) auto-correlation coefficient (AC), (e) cross-correlation coefficient (CC), and (f) The ratio of root mean square in each direction to the root mean square vector magnitude (RMSR). Velocity was calculated by the time taken for each 10-m walking test, and the mean of each value was used for the analysis. Cadence, defined as steps per minute, was calculated using the steps counted from the acceleration data and walking time. Step length was calculated using velocity and cadence. To compare the similarity of two different functions mathematically, a cross-correlation function was used. The cross-correlation function expressed the similarity of two functions as a value from -1 to 1. It used the convolution of functions to calculate the value. Especially, the value of the cross-correlation function of the two same functions was named the auto-correlation function. Each value of the auto-correlation function is a correlation coefficient between the raw acceleration data and the data to be shifted at some sample points from the same raw acceleration data. When the shifted sample point is 0, the position is defined as the reference point. A similar method to the auto-correlation function, called the auto-correlation method, was used in gait analysis [11, 12, 16]. From the processed acceleration data by the auto-correlation method, the first coefficient peak next to the reference point was defined as AC. The mean of 12 ACs was used for the analysis; this value represented step regularity [11, 12]. As for the AC, by selecting the acceleration data of every 6 pairs of the 12 walking trials (1st measurement and 7th measurement, 2nd measurement and 8th measurement, and so on), the value of the cross-correlation function of each data pair was calculated. The maximum value in the cross-correlation function of each pair was defined as CC. The mean of 6 CCs was used for the analysis. From the definition of CC, it represented gait repeatability. A proportional relationship has been shown between the root mean square of acceleration acquired from an accelerometer fixed on the body trunk and the square of gait velocity [9, 13]. To eliminate the relation between measured accelerations and gait velocity as much as possible, RMSR was proposed [17]. The same method was adopted in this study. The mean of 12 RMSRs was used for the analysis. The RMSR represented the degree of sway during gait [17].

### Statistical analysis

Statistical analysis was performed using R (Foundation for Statistical Computing, Vienna, Austria). The assumption of a normal distribution was assessed by the Kolmogorov-Smirnov test. The independence of the gender ratio between the patients and control subjects were assessed by the chi-square test. The difference of the mean age between the patients and control subjects was assessed by *T*-test for two independent groups. The difference in standing and gait parameters between both groups was assessed by the Wilcoxon rank sum test. The difference between the paired data during standing was assessed by the Wilcoxon signed rank test. The correlation between disease duration and the standing and gait parameters was assessed by Spearman’s rank correlation coefficient. The difference between the chronological changes in the control subjects was assessed by the paired *T*-test. The level of significance was set at *p* < 0.05 in all tests.
